# Usefulness of net retrieval devices for central airway obstruction caused by blood clots during extracorporeal membrane oxygenation: Case series

**DOI:** 10.1097/MD.0000000000039094

**Published:** 2024-07-26

**Authors:** Satoshi Tanaka, Nobuaki Yoshimura, Ryo Asakawa, Satoshi Tobita, Moto Yaga, Kiyonobu Ueno

**Affiliations:** a Department of Respiratory Medicine, Osaka General Medical Center, Osaka, Japan.

**Keywords:** central airway obstruction, extracorporeal membrane oxygenation, flexible bronchoscopy, net retrieval devices, severe respiratory failure

## Abstract

**Rationale:**

Extracorporeal membrane oxygenation (ECMO) is the last trump card for severe respiratory failure. The main complications of ECMO are bleeding and thrombosis, both of which can be life-threatening. Large blood clots can cause central airway obstruction (CAO) during ECMO, and CAO should be removed as soon as possible because of asphyxiation. However, there is no comprehensive reports on its frequency and management. The purpose of this study is to share therapeutic experiences for rare and serious conditions and provide valuable insights.

**Patient concerns:**

We report 3 patients placed on ECMO for severe respiratory failure.

**Diagnosis:**

CAO due to large blood clots occurred during ECMO in all 3 patients.

**Interventions:**

Large blood clots were removed using flexible bronchoscopy, grasping forceps, and net retrieval devices in all 3 patients.

**Outcomes:**

In all 3 patients, large blood clots were removed multiple times during ECMO. The patients’ respiratory conditions improved and they were eventually weaned off the ECMO.

**Lessons:**

CAO due to large blood clots during ECMO is rare. The frequency of CAO requiring bronchoscopic removal was estimated to be approximately 1,5%. When this occurs, clots should be removed as soon as possible. Net retrieval devices are useful tools for the collection of large blood clots.

## 1. Introduction

Extracorporeal membrane oxygenation (ECMO) is considered a highly effective treatment modality for severe respiratory failure.^[1]^ This method has been reported to be clinically effective for severe acute respiratory distress syndrome resulting from influenza,^[2]^ Middle East respiratory syndrome coronavirus,^[3]^ and coronavirus disease 2019^[4]^ infection. The main goal of ECMO is to achieve “lung rest,” and treatment of the primary disease should be maximized while the patient is in this ECMO-supported state. Major complications related to ECMO include bleeding and thrombosis, both of which may be life-threatening.^[1]^

Central airway obstruction (CAO) refers to airway obstruction in the trachea and main stem bronchi. The causes of this condition CAO are classified as malignant and nonmalignant.^[5]^ Malignant CAO occurs most often in lung cancer, whereas nonmalignant CAO occurs in granulation tissue resulting from endotracheal or tracheostomy tubes, foreign body aspiration, tracheobronchomalacia, and blood clots. CAO should be removed as soon as possible because it poses the risk of death by asphyxiation.

We encountered 3 cases of CAO caused by large blood clots during ECMO, in which the CAO was successfully released using flexible bronchoscopy, grasping forceps and net retrieval devices. No comprehensive reports have been published on the usefulness of net retrieval devices in removing CAO. Herein, we report the usefulness of net retrieval devices for collecting large blood clots.

## 2. Case presentation

This study was approved by the Ethics Committee of Osaka General Medical Center (No. 2023-060). Written informed consent was obtained from the patient and the patient next of kin to publish this report in accordance with the journal’s patient consent policy.

### 2.1. Case 1

**Figure 1.**
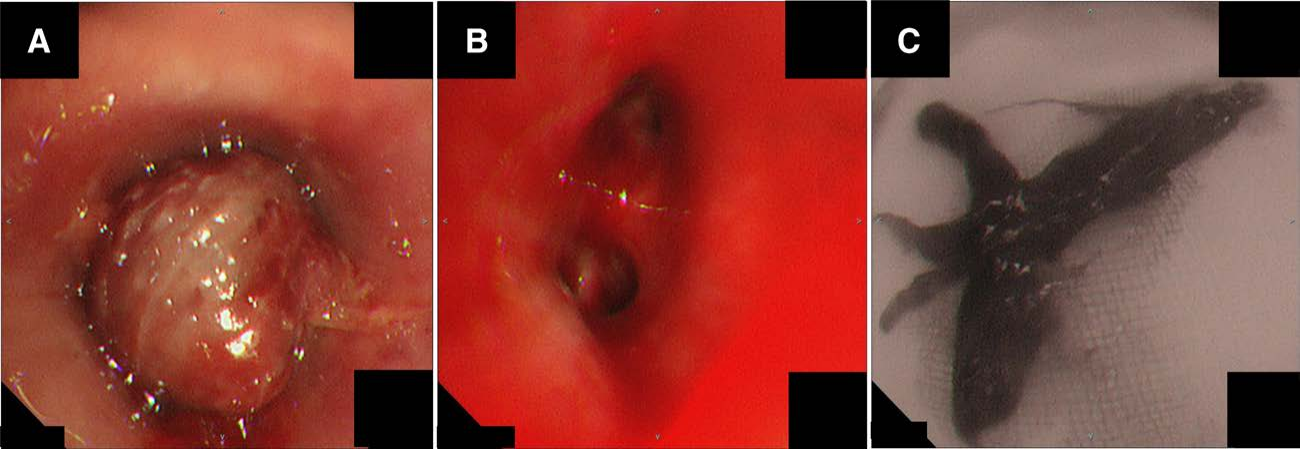
(A) Flexible bronchoscopy showed obstruction of the left main bronchus by a large blood clot. (B) Flexible bronchoscopy showed opening of the left main bronchus after clot retrieval. (C) A large cast-shaped blood clot with a long diameter of >7 cm was removed via bronchoscopy.

A 9-year-old boy was brought to our hospital via ambulance for high-energy trauma caused by a fall on the sixth floor. The diagnoses were bilateral pulmonary contusions, bilateral pneumothorax, multiple organ hemorrhages, and multiple fractures. The patient was intubated and placed on veno-arterial extracorporeal membrane oxygenation (V-A ECMO). Two days later, his tidal volume decreased to <50 mL; following this, he was referred to our department because of suspected CAO. Bronchoscopy revealed an obstruction of the left main bronchus by a large blood clot (Fig. 1A). Several blood clots were removed using flexible Olympus BF TYPE 1T290 and P290 bronchoscopes (Olympus, Tokyo, Japan), Radial Jaw ^TM^4 Standard Capacity grasping forceps (Boston Scientific, Marlborough, MA), and Rescue Net ^TM^ retrieval net (Boston Scientific, Marlborough, MA). First, the stem of the blood clot was detached using grasping forceps, while remaining careful to avoid bleeding. The detached blood clot was retrieved with a retrieval net and removed as the bronchoscope was withdrawn (Fig. 1B and C). Blood clots were removed twice over the 23-day ECMO period.

### 2.2. Case 2

**Figure 2.**
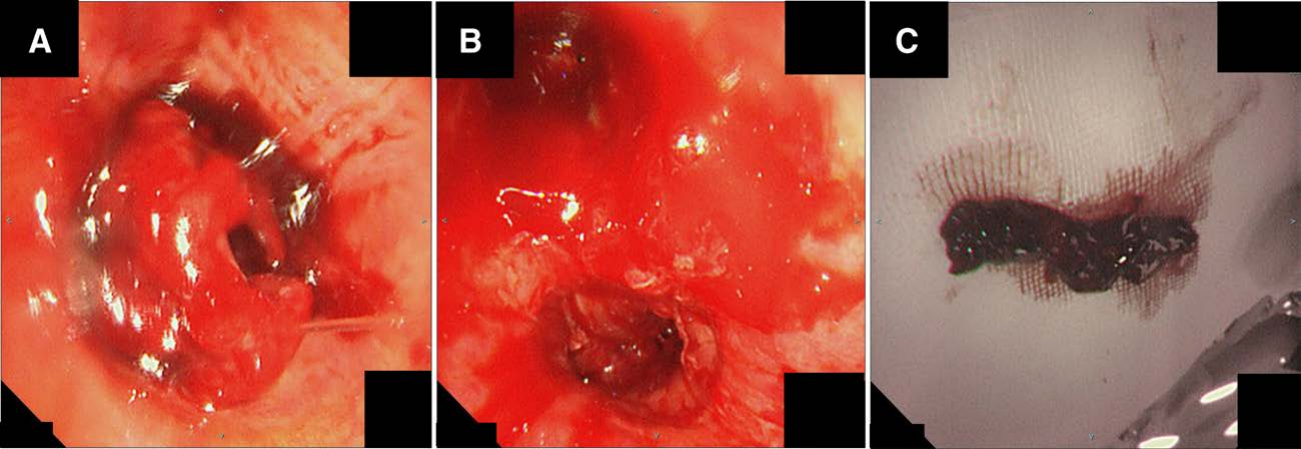
(A) Flexible bronchoscopy showed obstruction of the left main bronchus by a large blood clot. (B) Flexible bronchoscopy showed opening of the left main bronchus after clot retrieval. (C) A huge blood clot with a long diameter of >6 cm was removed via bronchoscopy.

An 85-years old woman underwent mitral valve replacement for severe mitral stenosis. Immediately following the surgery, the patient experienced massive bleeding from the chest drain and went into cardiopulmonary arrest. V-A ECMO was initiated and emergency surgery was performed. The patient’s condition gradually improved postoperatively. However, 42 days postoperatively, her respiratory status deteriorated again owing to aspiration pneumonia, and she was once again placed on veno-venous ECMO. At 44 days postoperatively, the patient developed small hemoptysis, and her tidal volume decreased to <100 mL. The patient was referred to our department for the examination of bilateral hemoptysis and CAO. Bronchoscopy revealed an obstruction of the left main bronchus by a large blood clot (Fig. 2A). Multiple blood clots were removed using the same instruments and methods as those used in Case 1 (Fig. 2B and C). Blood clots were removed five times over 15 days of ECMO induction.

### 2.3. Case 3

**Figure 3.**
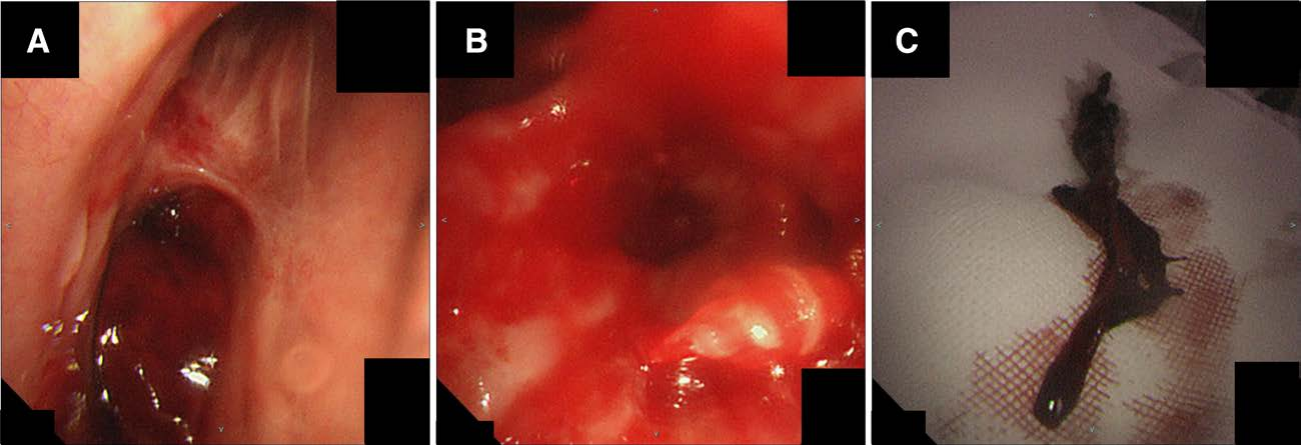
(A) Flexible bronchoscopy showed obstruction of the right middle trunk by a large blood clot. (B) Flexible bronchoscopy showed opening of the right middle trunk after clot retrieval. (C) A large cast-shaped blood clot with a long diameter of >7 cm was removed via bronchoscopy.

An 85-years old woman was brought to our hospital in an ambulance with chest and back pain. The patient was suspected to have acute myocardial infarction and was immediately intubated because of poor respiratory condition. Percutaneous coronary intervention for a 3-vessel lesion (#2:99%, #4PD:100%, #11:100%) was performed under ventilator control, following which V-A ECMO was initiated due to hemodynamic instability. Five days after initiation of ECMO, the patient developed small hemoptysis and her tidal volume decreased to <100 mL. The patient was referred to our department for the examination of suspected hemoptysis and CAO. Bronchoscopy revealed an obstruction of the right truncus intermedius by a large blood clot (Fig. 3A). Several large blood clots were removed using the same instruments and methods as those used in Cases 1 and 2 (Fig. 3B and C). Blood clots were removed twice over an 8-day period of ECMO induction.

## 3. Discussion

The major complications of ECMO are bleeding and thrombosis,^[1]^ which can be fatal and require immediate attention. In the literature, the estimated rates of bleeding and mortality as complications during veno-venous ECMO treatment are 29.3% and 6.9%.^[6]^ Flexible and rigid bronchoscopies are important cornerstones in the diagnosis and treatment of CAO. The goals of this treatment are airway patency and symptom relief by debulking or removing the obstructive material, possibly accompanied by stenting airway as well. Rigid bronchoscopy requires more complex techniques and more technical medical resources, while flexible bronchoscopy can be performed quickly and with fewer medical resources. The success rates of flexible bronchoscopic foreign body removal has been reported to be 90%.^[7]^ Various types of equipment, including grasping forceps, rubber-tip forceps, retrieval baskets, snares, balloon-tipped catheters, magnet-tipped probes, and cryoprobes, can be used to remove foreign bodies via bronchoscopy.^[8–10]^ The success rate of blood clot removal by cryoextraction has been reported to be 93%.^[11]^ Moreover, when removal of a foreign body proves challenging, combinations of these devices should be used. Net retrieval devices, for example, are useful for wrapping and removing entire tumors. In gastroenterology, these devices are routinely used to remove tumors resected by endoscopic mucosal resection (EMR) and endoscopic submucosal dissection.^[12]^ In our case, as the clots were relatively soft, we predicted that grasping the clot with alligator, tripod, or pentapod forceps would tear off some portions and complicate the collection of entire clots. The blood clots were quite large, and patients faced a significant risk of suffocation if torn portions of the clots entered the intubation tubes during collection. Therefore, it was necessary to collect the entire clot by pinching them with net retrieval devices. In all 3 cases in this report, removing large blood clots entirely using net retrieval devices enabled the release of the CAO. Therefore, we recommend net retrieval devices as highly useful tools for collecting large soft blood clots.

No comprehensive reports have been published on CAO caused by large blood clots during ECMO. We examined 209 cases of ECMO support performed at our center from April 1, 2019 to October 31, 2022, 3 of which resulted in CAO requiring bronchoscopic removal. The frequency was estimated to be approximately 1.5%. CAO due to large blood clots during ECMO is a rare complication. However, when this occurs, the clot should be removed as soon as possible.

## 4. Study limitations

Due to the small sample size, the findings in this single-case report remain to be validated by subsequent clinical trials with large sample sizes.

## 5. Conclusion

The incidence of severe CAO due to large blood clots requiring bronchoscopic removal during ECMO is estimated to be approximately 1.5%. When severe CAO due to large blood clots occurs during ECMO, immediate clot removal is desirable. Net retrieval devices are useful tools for wrapping and removing entire tumors, as well as large soft blood clots.

## Author contributions

**Investigation:** Nobuaki Yoshimura, Ryo Asakawa, Satoshi Tobita, Moto Yaga.

**Supervision:** Kiyonobu Ueno.

**Writing – original draft:** Satoshi Tanaka.
